# Breaking the vicious circle: Experiences of people in chronic pain on the pain rehabilitation journey

**DOI:** 10.1002/nop2.512

**Published:** 2020-05-29

**Authors:** Hafdís Skúladóttir, Thora J. Gunnarsdóttir, Sigríður Halldórsdóttir, Herdís Sveinsdóttir, Janean E. Holden, Amalía Björnsdóttir

**Affiliations:** ^1^ Faculty of Nursing School of Health Sciences University of Akureyri Akureyri Iceland; ^2^ Faculty of Nursing School of Health Sciences University of Iceland Reykjavik Iceland; ^3^ Faculty of Graduate Studies School of Health Sciences University of Akureyri Akureyri Iceland; ^4^ University of Michigan School of Nursing Ann Arbor Michigan USA; ^5^ School of Education University of Iceland Reykjavik Iceland

**Keywords:** chronic pain, illness, interviews, pain rehabilitation, phenomenology

## Abstract

**Aim:**

To explore the lived experience of individuals' in chronic pain of participating in a pain rehabilitation programme in Iceland.

**Design:**

Phenomenological research.

**Method:**

The Vancouver School of Doing Phenomenology. Eleven participants were interviewed.

**Results:**

The overarching theme was as follows: “the journey of breaking the vicious circle of chronic pain.” Before the programme, the participants felt they were in survival mode, trying to survive each day; they were stuck in a vicious circle of chronic pain, simultaneously trying to ease and conceal the pain. Reaching out for professional help was a turning point. While attending the programme, participants began deconstructing their old ways of dealing with chronic pain. After completing the programme, they were still reconstructing their daily lives. In conclusion, pain rehabilitation programmes can be the first step towards breaking the vicious circle of chronic pain.

## 1 INTRODUCTION

Chronic pain has been defined as pain lasting 3 months or more or as pain persisting beyond the time of expected healing (Treede et al., 2015). Chronic pain is a complex disorder interfering with all aspects of an individual's life (Sharpe, Alderson, & Collins, 2013), resulting in decreased physical activity (Boutevillain, Dupeyron, Rouch, Richard, & Coudeyre, 2017; McCracken & Gutiérrez‐Martínez, 2011), poor physical health (Macfarlane et al., 2009; Zanocchi et al., 2008) and insomnia (Aghayev, Sprott, Bohler, Röder, & Müller, 2010; Alföldi, Dragioti, Wiklund, & Gerdle, 2017; Canivet et al., 2008; Hamilton, Catley, & Karlson, 2007; Harman, Keating, Mayes, Walsh, & MacCallum, 2014). Qualitative studies show that chronic pain can influence the sense of self (Ahlsen, Mengshoel, & Solbrække, 2012; Biguet, Nilsson Wikmar, Bullington, Flink, & Löfgren, 2016; Osborn & Smith, 2006; Sharpe et al., 2013; Smith & Osborn, 2007) and psychosocial well‐being (Ojala et al., 2016), affect the ability to work (Andersen, Clausen, Burr, & Holtermann, 2012; Norrefalk & Borg, 2012; Stålnacke & Östman, 2010), result in job changes (Breivik, Collett, Ventafridda, Cohen, & Gallacher, 2006), strain finances (Andrews, Steultjens, & Riskowski, 2018; Norrefalk & Borg, 2012) and negatively affect family relationships (Ailshire & Burgard, 2012; Armentor, 2017). In Europe, the estimated prevalence of chronic pain is 12% (Breivik et al., 2006). In Iceland, the prevalence ranges from 19% (Bjornsdottir, Jonsson, & Valdimarsdottir, 2013) to 47.5% (Jonsdottir, Aspelund, Jonsdottir, & Gunnarsdottir, 2014). Despite the prevalence and serious consequences of chronic pain, there are no easy ways to treat it.

According to Axon, Patel, Martin, and Slack (2019) systematic review of population‐based studies, a substantial portion of community‐dwelling adults is likely to use prescription and non‐prescription medication for their pain along with non‐pharmacological strategies such as hot and cold packs and exercise. Multidisciplinary pain management interventions facilitate and support the development of individual self‐management strategies (Devan, Hale, Hempel, Saipe, & Perry, 2018). With professional individualized support, pain rehabilitation programmes can benefit the individuals' possibility of returning to work (Norrefalk & Borg, 2012).

Cognitive behavioural therapy (CBT), hypnosis (Castel, Cascón, Padrol, Sala, & Rull, 2012) and other mindfulness‐based approaches (Doran, 2014) are well‐known treatments used in pain rehabilitation programmes. CBT is based on the assumption that the way of thinking motivates behaviour and emotions (Sveinsdottir, Eriksen, & Reme, 2012). Combined with other treatments, CBT is a beneficial treatment for chronic back pain (Sveinsdottir et al., 2012) and fibromyalgia (Imamura, Cassius, & Fregni, 2009), which are the most common causes of pain among those who attend pain rehabilitation programmes (Gustafsson, Ekholm, & Ohman, 2004; Huet, Innes, & Whiteford, 2009; Merrick & Sjölund, 2009). CBT has been found to have long‐term effect on patients' pain management in their daily lives (Egan, Lennon, Power, & Fullen, 2017; Hållstam, Stålnacke, Svensen, & Löfgren, 2015).

## 2 BACKGROUND

When participants in pain management programmes are able to change their behaviour by changing their thoughts and feelings, they gain new insights and understandings (Haraldseid, Dysvik, & Furnes, 2014) and provide new skills to reduce pain levels and allow the participants to move towards a better life (Dysvik, Kvaløy, & Furnes, 2014). Qualitative studies focusing on the influence of pain rehabilitation programmes indicate that individuals with chronic pain acknowledge that accepting the persistency of pain is the way to move forward (Biguet et al., 2016). Moreover, using combined therapies in programmes has led to self‐healing with strength and a sense of well‐being (Gunnarsdottir & Peden‐McAlpine, 2004).

Even several months after using the intervention for self‐managing pain, the journey continues to be exhausting and a struggle (Devan et al., 2018; Hållstam et al., 2015). However, years later, these individuals still used the key strategies to manage their pain effectively after embedding them in their daily lives to improve their quality of life (Egan et al., 2017).

Research about pain rehabilitation programmes in Iceland has focused on CBT for depression and anxiety (Ólason, Andrason, Jónsdóttir, Kristbergsdóttir, & Jensen, 2018), patients' participation in their health assessment (Thorarinsdottir, Kristjansson, Gunnarsdottir, & Björnsdottir, 2019) and the use of a combination of complementary therapies (Gunnarsdottir & Peden‐McAlpine, 2004). However, to our knowledge, no previous study has evaluated the lived experience of participating in a pain rehabilitation programme.

Therefore, this study explores the lived experience of individuals' in chronic pain who participate in a pain rehabilitation programme. Participants were interviewed before and after the programme to increase knowledge and deepen the understanding of their lived experience over time. The goal of the study was to learn how patients experience their pain, health and well‐being before and after participation in the programme.

## 3 METHODS

### 3.1 Design

**FIGURE 1:**
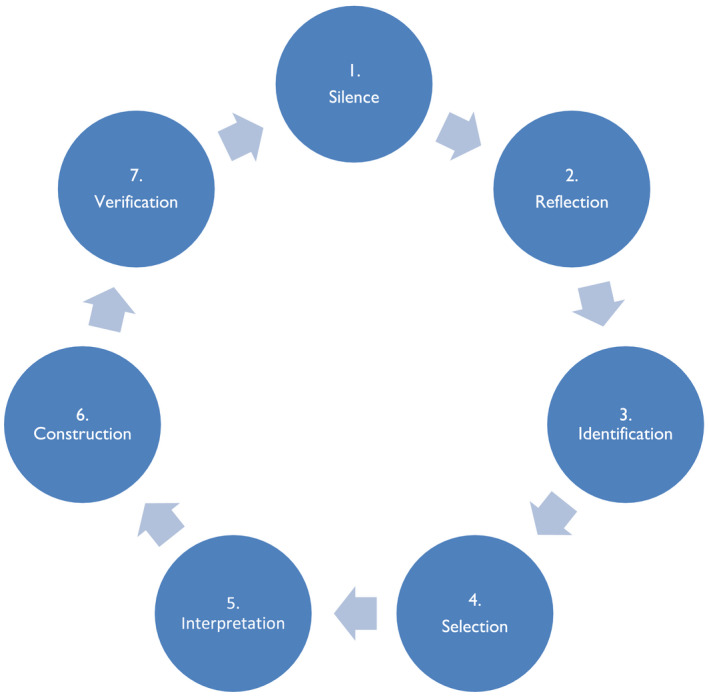
The process of doing phenomenology in the Vancouver‐School [Modified figure from Halldorsdottir (2000) p. 56. Used with permission]. This cycle is repeated in each of the 12 steps of the Vancouver‐School

The Vancouver School of Doing Phenomenology (in short the Vancouver‐School) was used in this study (Halldorsdottir, 2000). The qualitative approach used in this study offers a useful direction to nurse researchers because of its 12‐step approach (Dowling & Cooney, 2012), which has proven effective when used in the context of the lived experience of pain (Karlsdottir, Halldorsdottir, & Lundgren, 2014; Skuladottir & Halldorsdottir, 2011). This methodology is based on the works of Spiegelberg (1982) (phenomenology), Ricoeur (1980, 1981) (hermeneutic phenomenology) and Schwandt (1994) (constructivism). The Vancouver‐School is based on the philosophy of holism and existential psychology and on the premise that reality is individually constructed because of lived experience (Spiegelberg, 1982). In phenomenological research, the focus is on identifying and describing the common meaning several individuals have about their lived experiences related to a concept or phenomenon (Creswell, 2013). The Vancouver‐School has seven main cognitive aspects that are set up as a circular process and repeated throughout the research process: silence, reflection, identification, selection, interpretation, construction and verification (Figure 1). The implementation of the study was conducted in 12 main research steps; Table 1 shows how the steps were followed.

**TABLE 1:** Steps in the research process of the vancouver school of doing phenomenology

Steps	Description of each step	What was done in the present study
Step 1 The sample	Selection of participants who have experienced the phenomenon	The participants were recruited with collaboration from both chief physicians and head nurses at the rehabilitation sites who went through the waiting lists of incoming patients and compared it to the inclusion criteria. They then prepared a list of names and sent it to the primary researcher. This information was then used to contact potential participants by email
Step 2 Making pre‐conceived ideas visible	Preparation of the mind before the dialogues. Putting aside pre‐conceived ideas	The primary researcher reflected on own thoughts, pre‐understandings and pre‐conceptions about the phenomenon and kept a reflective journal
Step 3 Data collection	One or two interviews with each participant. Number of participants is decided when saturation has been reached	The interviews took place in locations of the participants' choice, in their homes (one), telephone interviews (thirteen) or at the primary researcher's office (seven)
Step 4 Beginning data analysis	Sharpened awareness of ideas and concepts. Data collection and data analysis runs concurrently	As soon as an interview began, the data analysis began as well and continued throughout the data collection period. At first, the text was read carefully, without coding. Then, the text was read several times and items were coded
Step 5 Individual theme analysis	Constructing the essential structure of the phenomenon for individual participants	Every transcript from each participant was read several times over to begin to construct the essential structure of the phenomenon according to each participant. Trying repeatedly to answer the question: What is the essence of what each participant is saying?
Step 6 Case construction	Findings developed for each participant	The main themes of interviews were highlighted, and the most important factors were used as building blocks for the individual case construction. An overview, or analytic framework, was constructed for each participant, and care was taken that they were fully consistent with the experience of that participant and the relevant research data
Step 7 Verification I	Confirmation of the findings with each participant	An overview of themes from the first and second interviews was prepared for each participant with first draft of structured themes: one from the first interviews and another structure from the second interviews. This was sent to each participant through email and asked for confirmation. Eight participants replied and sent their verification
Step 8 The overall findings	Ask repeatedly: What is the essential structure of the phenomenon?	After reviewing the individual case construction, the primary researcher constructed together with two co‐authors (SH and ThJG) one essential structure of the phenomenon of living with chronic pain before and after rehabilitation
Step 9 Verification II	The overall findings compared to the study data	The primary researcher reread all the transcript to make sure the interpretation was based on actual data and compared them with the essential structure of the phenomena
Step 10 Finding the essence of the phenomenon	Choosing the overall theme of the study that best describes the phenomenon	The name of the study is as follows: *The journey of breaking the vicious circle of chronic pai*n
Step 11 Verification III	Confirmation of the overall results with some of the participants	The overall findings were presented by the primary researcher to four participants who had attended one of the three pain rehabilitation programmes. They were satisfied with the results and verified them
Step 12 Writing the results	Multi‐voiced reconstruction to increase trustworthiness of the findings	The voice of all the eleven participants was included in the writing of the results, by quoting them directly. An effort was made to put the most important evidence from the data that best described the phenomenon and thus answered the research question

### 3.2 Settings

The study was conducted at the three rehabilitation centres in Iceland offering pain rehabilitation, which are referred to as Sites 1, 2 and 3. The staff members in all three rehabilitation centres include nurses, physicians, physiotherapists and psychologists and occupational therapists, social workers, nutritional consultants, massage therapists and physical activity instructors. Patients with chronic pain up to 70 years of age can attend Sites 1 and 3, but Site 2 only accepts patients aged up to 60 years (programme descriptions are presented in Appendix 1).

### 3.3 Participants

The Vancouver‐School requires 10–12 participants and 1–2 interviews per participant to obtain a minimum of 15 interviews (Halldorsdottir, 2000). The inclusion criteria for participating in the study were chronic musculoskeletal pain for at least 3 months; ability to speak, understand and read Icelandic; age 18–70 years; and being admitted to one of the three rehabilitation centres. Thirty‐three incoming patients received an introductory letter about the study inviting them to participate, which included information about the primary researcher, reasons for the study, the study goals and focus, the approximate lengths of the first and second interviews and the participants' ethical rights. Of the 33, 13 responded and 11 agreed to participate, which met the criteria for using the Vancouver‐School. Two refused to participate because of language difficulties or insufficient energy and 20 did not reply.

Participants applied for the pain rehabilitation programme after recommendation from their advisor in the vocational rehabilitation programme, their general physician (GP) or a specialist physician (Table 1: Step 1). The participants were aged 32–65 years (*M* = 47 years), with two male and nine female participants. Five were from Site 1, four were from Site 2, and two were from Site 3 (Table 2).

**TABLE 2:** Participants' description

Pseudonyms^a^	Age range^a^	Employment and family status	Pain sites‐diagnosis	Years in pain	Weeks in the programme
Anne	55–60	Unemployed, married, one child	Back pain	Two	Seven
Dave	30–35	Unemployed, unmarried, no child	Widespread pain, fibromyalgia, headaches and muscle spasm	Nineteen	Four
Eve	40–45	Working full‐time, divorced, four children	Most joints, knee, back pain, headache, Raynaud's and arthritis	Fifteen	Five
Helen	60–65	Unemployed, married, two children	Back pain	Four	Seven
Isabella	40–45	Unemployed, unmarried, three children	Back pain and fibromyalgia	Twenty	Five
John	30–35	Unemployed, married, three children	Gastrointestinal disease, arthritis unspecified, hip, feet, ribs and joints	Three	Six
Catherine	45–50	Working full‐time, unmarried, three children	Widespread pain, neuropathic pain in the upper part of the body and face, migraine, back pain and fibromyalgia	Fifteen	Four
Lena	55–60	Working part‐time, divorced, two children	Psoriasis arthritis, fibromyalgia, hand, feet, shoulder and back pain	Sixteen	Four
Maria	45–50	Unemployed, married, five children	Back pain, fibromyalgia, hip and shoulder pain	Twenty	Five
Rose	35–40	Unemployed, married, four children	Back pain, hands and fibromyalgia	Fifteen	Five
Sarah	55–60	Working part‐time. Cohabiting, four children	Back pain and fibromyalgia	Fifteen	Five
MEAN	47 years	Mostly married or cohabiting with children	Mostly back pain and fibromyalgia	13 years	5 weeks

^a^ To protect participants' anonymity.

### 3.4 Data collection and analysis

Data were collected through interviews. Initial interviews (11) were conducted before the participants attended the pain rehabilitation programme, and the second interviews (10) were conducted 3 months after they completed the programme. The first author (hereafter, the researcher) prepared an interview guide (Appendix 2) based on a critical literature review and discussion with the co‐authors and conducted all the interviews.

The interviews lasted from 22 to 80 min (mean = 37 min) and were audio‐recorded and transcribed verbatim, without including any information that could identify the participants. The participants were all given pseudonyms (Table 1: Steps 2 & 3).

Every interview was conducted with an open mind because each person had a unique story to tell. In the second interviews, the researcher presented the data analysis of the participants' first interviews. This approach was used to help the participant to compare their lived experience of pain, daily life, health and expectations before the rehabilitation to the lived experience during the pain rehabilitation programme and the time after completing the programme. This approach was also done for verification. As more interviews were conducted, the researchers realized the nature of the phenomenon in more depth. New information was obtained that allowed the researcher to delve deeper into aspects of the phenomenon, to ask more detailed questions about relevant aspects and to determine the factors that were irrelevant to the phenomenon (Table 1: Steps 4–6).

When conducting the 19th interview from the primary sample of 10 participants, the start of data saturation became evident. After obtaining additional data from one more participant, it was determined that enough data had been obtained to answer the research question.

NVivo 11 (QSR International) qualitative data analysis software was used to manage the dataset and for within‐ and between‐case comparisons. At each step in the data analysis, the researcher analysed the transcription for themes according to the Vancouver‐School protocols (Figure 1). The findings from each participant were constructed into an individual analytical framework (Table 1: Step 6) and verified by eight participants (Step 7). With two co‐authors, the essential structure of the phenomenon was constructed (Step 8) and verified (Steps 9–11). The voice of all participants was included in the findings by quoting them directly to increase the trustworthiness of them (Step 12). We adhered closely to the Consolidated Criteria for Reporting Qualitative Research (COREQ) 32‐item checklist reporting the methods, analysis and results of this study (Tong, Sainsbury, & Craig, 2007).

### 3.5 Ethical considerations

Permission to conduct the study was granted by The National Bioethics Committee (VSN‐15‐10) and chief physicians at the three rehabilitation centres.

All participants were offered postinterview support from a clinical psychiatric nurse specialist; however, no one used this option. The participants signed their informed consent and were guaranteed confidentiality.

## 4 RESULTS

**FIGURE 2:**
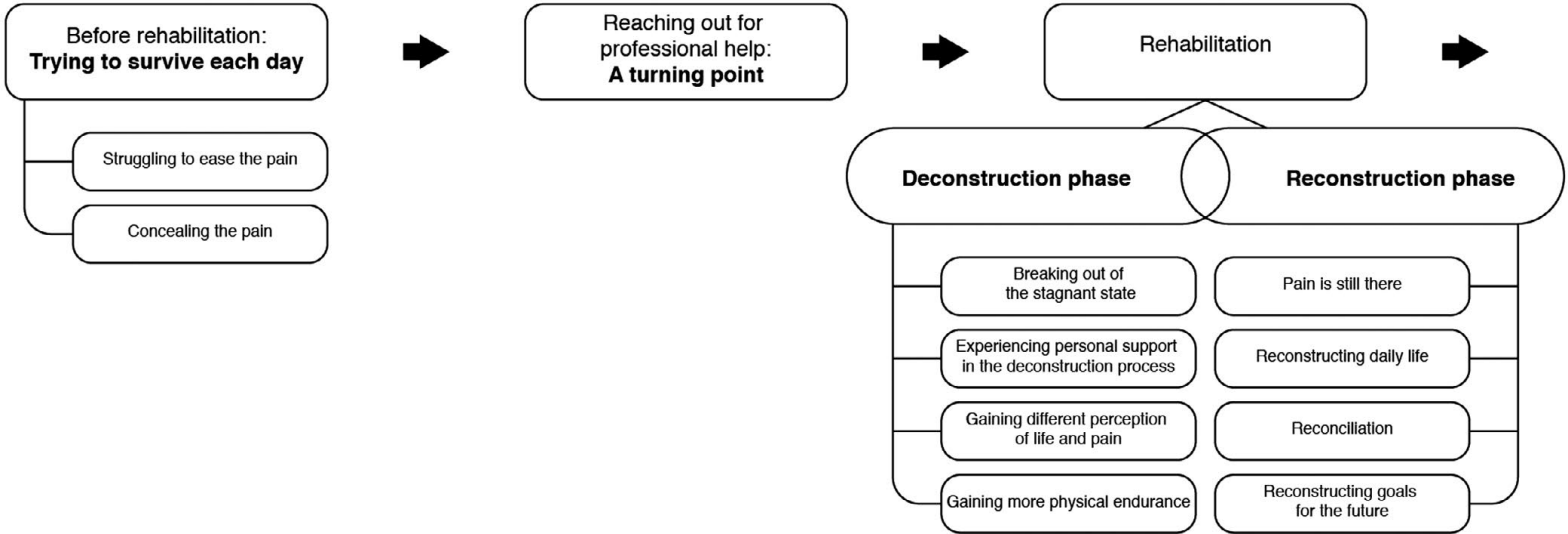
The Journey of Breaking the Vicious Circle in Chronic Pain: Overview of the study findings

The overarching theme of the study was as follows: “the journey of breaking the vicious circle of chronic pain,” which captures the essence of the participants' lived experience. Before attending the programme, the participants described themselves as being in a vicious circle of pain, trying to survive each day. After the programme, they described their journey of breaking that circle in rehabilitation and deconstructing their old ineffective ways of dealing with their chronic pain. Three months after completing the programme, the participants were still rehabilitating. However, they were no longer struggling to survive; they had started reconstructing their daily life and were more in control of their pain and starting to make goals for their future (Figure 2).

### 4.1 Before rehabilitation: trying to survive each day

The participants reflected on their daily pain, which fluctuated in magnitude from one day to another. The pain controlled their daily life and they struggled to find ways to ease the pain. Several participants described how they were stuck in a vicious circle. Some feared their future, not knowing where their situation would lead, and they feared losing their health. Part of their experience with the vicious circle was the difficulties they experienced falling and staying asleep because of the pain, worries, anxiety, uncomfortable bed and lack of understanding from others. Eve described it in this way: “I am not able to sleep, no matter what I do. I believe I am in some vicious circle. It has been like that for a long time.” Being able to get some rest and sleep through the night was important because sleepless nights meant more pain the following day. Several participants could not continue working because of their pain but dreamt of being able to return to work someday. They tried to survive each day without setting goals for the future. As Lena said: “I have no goals. The only goal I have these days is just surviving each day.”

#### 4.1.1 Struggling to ease the pain

The participants had tried pain medication but experienced little or no relief. Catherine described this problem: “No ordinary pain medication can relieve this pain… and it is difficult to distract your thoughts away from it.” Isabella shared:

I had been going to a physical therapist once a week for more than a year and nothing worked…. Then I went to [an orthopaedic surgeon] for injections twice and that did not work and he just wished me all the best; he could not do anything else for me.

Relaxation, massage, acupuncture, reflexology, heat, physical therapy, regular exercise, walking and hydrotherapy were some of the methods participants used to try to ease their pain. Keeping an open mind and engaging in positive thinking were reported to be helpful:

If you wake up one morning and decide that this will be a miserable day, then the day will be miserable. I tell myself that I am willing to try everything with an open mind. The worst that can happen is that nothing happens, and I will be at square one. (Isabella)

Distraction was another useful strategy. Reading and listening to music, participating in volunteer work and having a job helped to distract thoughts away from the pain: “It is the best, the best relaxation that I know of. That is either just lying down completely relaxing in the swimming pool, just letting myself float or just lying down with some music on” (Dave).

#### 4.1.2 Concealing the pain

In general, participants experienced that people closest to them realized what they were going through, but they did not always show concern. Other people did not understand why people who had no obvious problem could not have a job and do their “duties.” The participants concealed their pain, avoided talking about it to others and said that they were feeling good even if they were not: “I do not like to talk about it [the pain]. I do not need pity from others, so I have just learned to live with it and have stopped talking about it” (Eve).

### 4.2 Reaching out for professional help: a turning point

At a certain point, the participants realized that they were no longer able to take care of their situation. They had reached a stagnant state where nothing was changing, the strategies they used were ineffective and they felt they needed help. They therefore searched for and found a health professional who suggested a pain rehabilitation programme.

When the application had been sent, the participants suddenly experienced some hope that something could change for the better. They did not know exactly how they would benefit from it, but they were excited about starting the pain rehabilitation programme. Some were hoping to get answers, a diagnosis and increased physical endurance. Others were hoping for some “me time” for several weeks where they could focus on their health and well‐being or learn new strategies to live with the pain. No one expected to become completely pain‐free. Eve stated: “I need to learn some methods to ease the pain and exercise and strengthen myself so I can continue from that. So, I can feel better.”

### 4.3 Rehabilitation: deconstruction and reconstruction phase

As demonstrated in Figure 2, rehabilitation is a dynamic process of deconstruction and reconstruction.

#### 4.3.1 Deconstruction phase

##### Breaking out of the stagnant state

The participants were ready to be in the pain rehabilitation programme because the programme allowed them time to focus entirely on themselves and on enhancing their health instead of managing their daily routines where their focus was usually on the needs of others. Having children to take care of took much of the participants' effort and energy. The participants who had to go home in the afternoons or for a few days in the middle of the programme to take care of domestic chores and children experienced increased stress levels and they felt they gained less from the programme than they could have:

I am going to focus on this [the programme], focus on me, think only about me, not the needs of others, or doing something else. It will be my time to try to move on. If I cannot take care of myself then I cannot take care of others. (Isabella)

##### Experiencing personal support in the deconstruction process

Experiencing personal support from family, friends and co‐workers while at the rehabilitation centre was valuable to the participants. They could focus on themselves and their needs during the difficult deconstruction process. In all the three rehabilitation centres, the participants described the professional demeanour of the healthcare providers. The healthcare providers were caring, warm, flexible and eager to find the best schedule, for everyone: “The staff should be rewarded for their existence…. You always experienced so much warmth from everyone” (Rose).

Helen regained belief in people after staying in the pain rehabilitation programme. Anne said that her belief in the healthcare system improved after watching how other people regained their health in rehabilitation. The participants felt understood and respected by the staff, listened to and accepted; they were not just numbers but people who needed help with their problems:

I was in self‐destructive mode. Angry… I am most grateful for how [the health professionals] helped me to keep my family. I was losing them. They helped me to keep what was most precious to me. I did not arrive as some number to go through some conveyor‐belt and then be thrown out. We got deep into it… it was personal… they helped me. I did not expect that. (John)

Some of the participants experienced personal support, acceptance and understanding from their group as well. The group members were described as kind, understanding and caring. They showed empathy, tolerance, encouragement and positivity towards each other.

##### Gaining a different perception of life and pain

The programme helped the participants to gain new perspectives on their lives and pain, on how to think about and ease the pain and how to prevent pain attacks. Rose compared the chronic pain to a passenger whom she was trying to move to the backseat, presumably where it had a less disturbing impact than it did on the front seat.

When John started the programme, he was angry, and he was convinced that the doctors had made a mistake. His goal was to get out of the patient role: “I was always feeling sorry for myself… nothing was my fault, always someone else's fault…. I am learning to deal with it myself and learning to do it myself” (John).

Cognitive behavioural therapy, ergonomics, body awareness, massage, relaxation, stretching, hydrotherapy, shock wave therapy, mud bath and hand waxing were valuable and helpful strategies. For one participant, the hydrotherapy was the most valuable, while for others, the shock wave therapy or physical exercises were most valuable. Some participants felt they had not heard anything new in the lectures, yet they appreciated them. The lectures about pain and pain management were described by Isabella as an acceptance of the pain's existence: “Someone else knows what it's like which is an acceptance [of chronic pain] and [proves it is] not some hysteria. Sometimes pain cannot be described, but some people have it and such pain is individual” (Isabella).

##### Gaining more physical endurance

Physical exercises helped to improve participants' physical well‐being and enabled them to move more: “It strengthened me somehow and made me realise the state I was in when I attended [the programme] and helped me work through that. Now I respond differently to difficult periods” (Anne):

Sarah described how exhausted she was after the physical exercises and thought she was going to die and yet the programme made her realize that physical exercise was something that she needed to do every single day to feel better. She had more drive, and her health was better because of the physical exercises.

#### 4.3.2 Reconstruction phase

For most of the participants, a reconstruction process started while they were still in the programme:

It [the programme] is like a jigsaw and I have found a lot of puzzles.... I used to focus on one specific thing which was supposed to salvage me, but then it didn't. I was always trying to find the solution. But maybe combined, it [the treatments in the programme] created the solution. (Lena)

##### The pain is still there

The pain was still there, and the participants accepted that it was unlikely to go away. However, it no longer controlled everything in their daily lives and the disturbing effects on their daily lives were less than before. Mentally, something changed for the better: “Physically, it [the programme] did not change much… for the pain, it did not change much. However, mentally, I received more than I expected” (Rose).

##### Reconstructing daily life

Each participant had started to reconstruct their daily life by putting together the pieces they had experienced in the pain rehabilitation programmes that were most useful and suitable for them. The priorities in the participants' daily life had changed. They were focused on making more space for regular physical exercises, physiotherapy, rest, breaks at work and relaxing. These strategies improved their well‐being:

I am more aware of doing something for myself. I am not supposed to be left out. Not enforcing myself so much that I will be worse and worse and worse. I need to stop for a minute and think about myself. I need to take a break at work which I wasn't used to doing. Thinking back, I used to sit at work the whole day, just working. (Eve)

##### Reconciliation

The participants accepted themselves and their pain. They accepted their existence and realized that they no longer needed to defend their existence, as if they had been a burden on their family and society previously. They stopped making excuses for their existence, and they no longer needed a job title to define who they were:

When you start to accept yourself, it's like a snowball that starts rolling. I have stopped making excuses regarding why I am the way that I am. I have stopped using the job title when I define myself. I have reached a point where I do not need to defend myself anymore. (Rose)

Lena described how she started to define herself differently:

I started to write down what I was thinking…. I realised that I do not know who I am. I used to be so occupied with fitting into some form. I needed to stop being angry and I wasn't satisfied with that at the beginning because I had been angry for so long.

After she stopped being angry, she reconciled with her family. John described how he was at peace with himself and had learned to enjoy the moment:

I am living the dream I used to dream before I got sick. I am experiencing the balance with my family, with my life. My focus is on enjoying the moment because you never know when the next pain attack will strike.

##### Reconstructing goals for the future

By the second interview, the participants had not reached the end of their reconstruction process, but they were on their way. Their focus had changed to making goals for the future instead of regretting the past. “I am not going to spend the rest of my life thinking: ‘What could I have done differently’?” (Lena).

Rose described how she needed to focus more on preparing herself: “I am determined to do things. I know I must prepare myself. I am finding out how I can prepare myself according to what I am going to do. I am getting there.” The experience of taking the time to attend a pain rehabilitation programme and to focus on themselves and their needs made the participants realize that they needed to allow themselves time to get away from all the stress in their daily life and spend some time elsewhere.

## 5 DISCUSSION

Interviewing the participants before and after completing the programme provided valuable insights into the programme's influence on their thoughts about their pain and daily activities. In the second interviews, the participants could compare their situation at the time of the interview to where they were before they participated in the programme. The findings showed how their priorities had changed, how their focus was more on their well‐being and how the pain no longer dominated their life. Three months after completing the programme, they were still combining pieces they had experienced in the programme into a more holistic structure without knowing when and how their journey would end. They were trying to enjoy the moment of well‐being while it lasted and had started to reconstruct their life.

### 5.1 Changed priorities

Before attending pain rehabilitation, the participants were just trying to survive each day, struggling to ease the pain and they felt stuck in a vicious circle of chronic pain. However, in line with findings by other researchers (Egan et al., 2017; Hållstam et al., 2015), change was possible and the participants noticed positive changes. Three months after they completed the programme, their priorities had changed. They were going through changes, thought differently about themselves and were slowly making changes in their daily lives, as seen in another study (Egan et al., 2017). They had stopped making excuses for their existence and being angry, and they accepted themselves and their pain. As seen in previous studies (Hållstam et al., 2015), the participants received valuable support from their family, friends and co‐workers while in the rehabilitation programme. They thought more about how they could put their needs at the forefront and the attitudes of those with jobs towards their work environment changed. Similar to the findings of Gustafsson et al.'s (2004) study where rest was not possible or permitted before the programme, they became more aware of the importance of resting, both at work and at home, making it more possible and frequent after completing the programme.

As found in other studies, the participants started to make space for regular physical exercise (Hållstam et al., 2015), physiotherapy, relaxation (Gunnarsdottir & Peden‐McAlpine, 2004) and pacing (Egan et al., 2017; Hållstam et al., 2015). They managed to break the vicious circle where they were stuck before and began to reconstruct their lives. They were no longer only surviving; they were starting to live a life (Hållstam et al., 2015). As the findings of other research suggest (Doran, 2014; Dysvik et al., 2014; Egan et al., 2017; Haraldseid et al., 2014), it is possible that the CBT and mindfulness‐based approaches used in the pain rehabilitation programmes had an effect on their new ways of living.

### 5.2 Moving pain in the backseat

As found in another study (Hållstam et al., 2015), after completing the programme, the participants realized that their pain was permanent; it was a part of their life, so it was better to learn to live with it (Biguet et al., 2016). They had, however, stopped concealing their pain. They experienced more physical endurance and mental changes as well. They deconstructed their old and ineffective ways of dealing with their chronic pain and reconstructed new ways of thinking and living. Skills, such as non‐pharmacological treatment, hydrotherapy, pacing and physical exercises, to reduce pain and handle life, facilitated the change process as seen in other studies (Egan et al., 2017; Hållstam et al., 2015). The recovery was not one specific thing, it was several pieces combined, similar to Gunnarsdottir and Peden‐McAlpine's (2004) findings and we found that the combination of multiple complementary alternative therapies was crucial in the participants' healing. They needed help and guidance to learn new strategies. Participants also indicated that receiving acceptance and understanding from group members and healthcare professionals who empowered them to take responsibility in their daily lives and such empowerment has been reported in other studies (Biguet et al., 2016; Egan et al., 2017; Gunnarsdottir & Peden‐McAlpine, 2004; Hållstam et al., 2015). Here, it was also clear that they were guided into a new mode of being and of no longer letting the pain dominate their life.

### 5.3 Rehabilitation continues after the programme's completion

Three months after the programme's completion, we found that the participants were just starting to make changes and trying to realize the best ways to put their most valuable strategies they had learned in the programme into their daily routine.

The participants in the current study described the existence of a pain rehabilitation programme as recognition of their chronic pain. Applying for such a programme was a turning point in the participants' chronic pain trajectory, which they considered the first step in the process of breaking the vicious circle of chronic pain and of their stagnant state. They were hopeful that something would change for the better after completing the programme. No one expected to become completely pain‐free similar to the results of Geurts et al.'s systematic review (2017) where the patients in the papers studied expressed a want or a need for pain relief or pain cure but predicted substantial less pain relief or no pain reduction at all.

For how long does the positive influence of rehabilitation continue? Several previous studies have examined this question. For example, there is a possibility that some chronic pain sufferers who attend a pain rehabilitation programme return to survival mode instead of continuing to rehabilitate because the sustained effort of self‐managing chronic pain can be exhausting and motivation can wane over time following an intervention (Devan et al., 2018).

Therefore, it is perhaps worth implementing a follow‐up newsletter, refresher course, app (Egan et al., 2017), booster sessions and/or peer support groups (Devan et al., 2018) several weeks or months after the programme's completion. Additionally, a hotline and/or chatroom (online) could be set up to offer professional counselling and support.

### 5.4 Strength and limitations

A strength of this study is that the participants attended three different programmes.

The number of participants and interviews is well within limits described in phenomenological studies. However, it is impossible to say whether more participants would have further increased our understanding of the phenomenon. Despite the effort made to obtain secondary interviews with all participants, one of the 11 participants did not reply to the messages sent to plan the second interview and only eight participants verified their individual analytical framework. Another potential limitation of the present study is the time between programme completion and the second interview. Three months may not be long enough to fully understand the process and progress the participants were making. Their reconstruction was not completed by 3 months, and future studies should examine participants outcome over a year or longer.

## 6 CONCLUSIONS

The impact of chronic pain is multifaceted. Pain rehabilitation can assist sufferers to confront the pain, deconstruct unhelpful ways of dealing with pain, gain a different perspective about the pain and learn new ways to reconstruct daily life.

The results provide a deeper understanding of the impact of a pain rehabilitation programme and indicate what matters the most for the participants, which can be valuable for the future planning and development of these and similar programmes.

## CONFLICT OF INTEREST

None.

## AUTHOR CONTRIBUTIONS

All authors contributed to the study design (HS, TJG, SH, HS, JH, AB). The first author (HS) conducted the interviews. HS, TJG, SH and HS contributed to the data analysis and the drafting of the manuscript. All authors critically revised the manuscript.

## ETHICAL APPROVAL

Permission to conduct the study was granted by The National Bioethics Committee (VSN‐15‐101) and chief physicians at the three rehabilitation centres. The participants signed informed consent and were guaranteed confidentiality.

## APPENDIX 1

### Overview of the programmes in the three pain rehabilitation centres

**Table:**

Site 1	Site 2	Site 3
**Initial assessment**: Four days pre‐programme intended for interviews, evaluation and education. Goal setting and a decision is developed for further rehabilitation. People go home with a plan, for example to decrease their medication and increase exercise. After that first meeting, some patients start to change their lifestyle. Possibility of follow‐up before the standard programme begins **The standard programme** mainly focuses on individualized schedule for lifestyle changes, assessment, education, improving physical condition, CBT, compassion‐focused therapy (CFT), body awareness training, mindfulness, changing self‐image, relaxation techniques, sleep disturbances, psychiatric consultation**,** increasing the ability to cope with pain and minimizing or reducing pain medication consumption and increasing the ability to get back to work **Teaching** is both on an individual basis and in groups **Schedule:** The patients stay at the rehabilitation centre only during the daytime from 8 o'clock am to 4 p.m. for 5 to 7 weeks	**Initial assessment**: One day pre‐interviewing for evaluation and education. A meeting is held with the patient, a group of healthcare professionals and preferably with a family member **The standard programme** mainly focuses on cooperation between patients, their families and healthcare professionals, aimed at assessment, education, improving physical condition, CBT, body awareness training, changing self‐image, relaxation techniques, sleep disturbances, psychiatric consultation**,** increasing the ability to cope with pain and minimizing or reducing pain medication consumption and increasing the ability to get back to work **Teaching** is both on an individual basis and in groups **Schedule**: The patients stay at the rehabilitation centre 24 hr for 5 days a week for 4 weeks. Follow‐up week 3 months later	**Initial assessment**: One day where each individual/group is assessed in order to develop an individualized programme **The standard programme** mainly focuses on interviews, consultancy, assessment, observation and support, education, physiotherapy, rest and relaxation classes, where the focus is on the individual. Special emphasis is on daily mindfulness meditation, Mindfulness‐based cognitive behaviour therapy (MBCBT), CFT and body awareness (tai chi), which can help individuals in chronic pain to learn to know their own limitations and coping behaviour. Also offered are water exercises, health (herb) baths, mud baths, hot and cold packs, daily use of the swimming pool, jacuzzies and sauna, massage and acupuncture, and Kneipp water therapy **Teaching** is both on an individual basis and in groups **Schedule**: The patients stay at the rehabilitation centre 24 hr, 7 days a week for 4 weeks

## APPENDIX 2

### Interview guide. Main questions and examples of follow‐up questions

**Table:**

Questions before participants attended the pain rehabilitation program:	Questions at least 3 months after participants' programs' completion:
Can you describe the pain you have today, your health and your daily life?	Can you describe the pain you had before you attended the pain rehabilitation program and compare it to the pain you have today; Can you compare your health and life today to your health and life before you attended the pain rehabilitation program?
**Examples of follow‐up questions:**	**Examples of follow‐up questions:**
Background information Onset of pain Causes of pain Diagnosis Possible changes in relationships with family and friends Self‐image/how do you describe yourself? What affects your daily physical, psychological, social, and emotional well‐being and daily activities?(e.g. physical condition, food/beverages, environment, stress, sleep, insecurity, fatigue, job, disability, exercises, support/lack of support from family, friends, healthcare staff, people with pain and community, worries, isolation, domestic chores, distraction, leisure activities, education, access to healthcare, communication with healthcare staff etc) Reaction and resources regarding pain Roles within the family and abilities to do domestic chores Something you must deny yourself because of pain? Something that brings pleasure? Reasons for applying for rehabilitation program? How did that happen? Did you prepare yourself in some way before attending the program? Expectations regarding the program Is there something I have not asked you about, that you would like to tell me because you feel it matters?	Compare the resources you had before to relief the pain to the ones you have now. Has there been any changes? Can you describe other impacts on psychological well‐being, social activities, and leisure activities? (E.g. needs, resources, work abilities, job, roles within the family, support, sleep, worries, relaxation, more/less social activities, easier to express feelings? Etc.). How do you describe yourself? Has it changed after completing the program? Did the program meet the expectations you had? If so, in what way? Can you describe the treatment you received? Can you describe the education you received? Has there been any changes in what affects your daily physical, psychological, social, emotional well‐being and daily activities? (e.g. physical condition, food/beverages, environment, stress, sleep, insecurity, fatigue, job, disability, exercises, support/lack of support from family, friends, healthcare staff, people with pain and community, worries, isolation, domestic chores, distraction, leisure activities, education, access to healthcare, communication with healthcare staff etc) Is there something you would like to tell me, something I have not asked you about, but you feel it matters?
